# Supplementary material for: The Evolution and Characterization of the RNA Interference Pathways in Lophotrochozoa
Source: Genome Biol Evol. 2024 May 7;16(5):evae098. doi: 10.1093/gbe/evae098 (PMC11114477; doi:10.1093/gbe/evae098)
Supplement: evae098_Supplementary_Data [file evae098_supplementary_data.zip › Supplementary_materials.zip!/selected_miRNAs/NoGe_selected_miRNAs/known_miRNA_families_pdf_files/NMRB01003515.1_Notospermus_geniculatus_strain_Ushimado_scaffold3515,_whole_genome_shotgun_sequence_43828.pdf]

Provisional ID	:	NMRB01003515.1_Notospermus_geniculatus_strain_Ushimado_scaffold3515,_whole_genome_shotgun_sequence_43828
Score total	:	156656.2
Score for star read(s)	:	3.9
Score for read counts	:	156647.5
Score for mfe	:	1.9
Score for randfold	:	0
Score for cons. seed	:	3
Total read count	:	307269
Mature read count	:	307235
Loop read count	:	0
Star read count	:	34

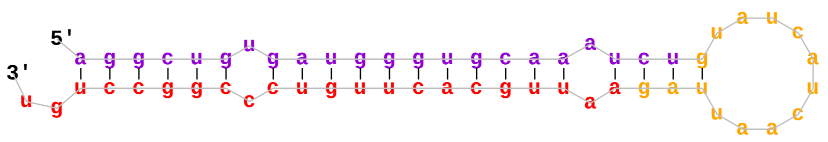

Genome coordinates: NMRB01003515.1 1929-1987

pre-miRNA sequence:
AGGCUGUGAUGGGUGCAAAUCUAGGCUGUGAUGGGUGCAAAUCUGUAUCAUCAAUUAGAAUUGCACUUGUCCCGGCCUGU

Mature sequence: AAUUGCACUUGUCCCGGCCUGU

Aligned mature sequence of the *N. geniculatus* pre-miRNA with the top 5 hits from the BLAST search against the pre-miRNA mirGeneDB set:

```
                              *********************
Nge_Mir-92-P1a               aauugcacuugucccggccugu
Rno-Mir-92-P1a_3p            uauugcacuugucccggccugu
Sha-Mir-92-P1a_3p            uauugcacuugucccggccugu
Xla-Mir-92-P1a4_3p           uauugcacuugucccggccugu
Xla-Mir-92-P1a3_3p           uauugcacuugucccggccugu
Xtr-Mir-92-P1a_3p            uauugcacuugucccggccugu
```
